# Responses of different functional tests in candidates for bariatric surgery and the association with body composition, metabolic and lipid profile

**DOI:** 10.1038/s41598-021-02072-x

**Published:** 2021-11-24

**Authors:** Paula Angélica Ricci, Larissa Delgado André, Soraia Pilon Jürgensen, Claudio Ricardo de Oliveira, Fernando Pinheiro Ortega, Luciana Di Thommazo-Luporini, Audrey Borghi-Silva

**Affiliations:** 1 Cardiopulmonary Physiotherapy Laboratory, Federal University of Sao Carlos – UFSCar, Rod. Washington Luis, km 235, Sao Carlos, 13565-905 Brazil; 2 Department of Medicine, Federal University of Sao Carlos, Sao Carlos, SP Brazil; 3 Ortega Clinic, Sao Carlos, SP Brazil

**Keywords:** Cardiology, Diseases, Endocrinology, Health care, Signs and symptoms, Metabolism

## Abstract

Individuals with obesity can have metabolic disorders and may develop impairments that affect the ability to exercise. The maximal incremental cardiopulmonary exercise test is widely used to assess functional capacity. However, submaximal tests such as the two-minute step test (2MST) and the six-minute walk test (6MWT) also allow this assessment. We propose to analyze whether body composition, metabolic and lipid profile influence the maximal and submaximal performance, and investigate these variables in response to different functional tests. Forty-four individuals with obesity, aged 18–50 years, underwent analysis of body composition, metabolic and lipid profile, incremental treadmill test (ITMT), 6MWT, and 2MST. One-way ANOVA, Pearson or Spearman correlation, and Stepwise multiple linear regression analysis were performed. ITMT induced a greater metabolic, ventilatory, cardiovascular, and perceived exertion demand when compared to the 6MWT and 2MST (p < 0.05). In addition, 2MST elicited a higher chronotropic (HR) and metabolic (V̇O_2_) demand when compared to the 6MWT (p < 0.05). Significant correlations were found between tests and body composition, metabolic and lipid profile. Fat mass and low-density lipoprotein can explain 30% of the V̇O_2_ variance in the ITMT; and fat mass, glucose, and performance in the 2MST can explain 42% of the variance of the distance walked in the ITMT. Obesity and its metabolic impairments are capable of influencing responses to exercise. ITMT generated greater demand due to the high stress imposed, however, 2MST demanded greater metabolic and chronotropic demand when compared to the 6MWT.

## Introduction

Obesity is a global^1^, chronic, and multicausal disease, causing disabilities and physiological changes in individuals^2^. In addition, obesity is related to several comorbidities that impair health^3^, and the deficiencies caused by being overweight may be attributed to chronic systemic inflammation^4^, insulin resistance, and disorders of lipid metabolism^5^, concurrent with decreased functional capacity^6^, and quality of life^7,8^.

Individuals with obesity have body composition changes, more specifically, increased body fat^9,10^, and may also present metabolic and lipid profile changes^11^. In addition, individuals with obesity and metabolic disorders may have greater limitations when an external stimulus is applied, such as physical exercise, and less efficient adaptive adjustments are made, leading to less functional capacity^12^. Some authors demonstrate that low functional capacity is independently associated with negative outcomes, such as the increased risk of mortality^13,14^.

The maximal incremental cardiopulmonary exercise test is the gold standard for assessing functional capacity^15^ and is also a test that holds an important prognostics value in the preoperative period of bariatric surgery^16^. Nevertheless, alternative tests to assess physical capacity are also feasible, making this assessment more accessible, since cardiorespiratory and metabolic adjustments can also be observed through submaximal field tests, which may be applied in the mediate postoperative period of bariatric surgery. Moreover, submaximal tests are important to assess the impact of intervention programs, in addition to being able to reveal functional capacity limitations in individuals with obesity. The submaximal tests have the characteristic of being less costly, in addition to enabling and interesting because they resemble activities of daily living^17^. The six-minute walk test (6MWT) being a test commonly used for different populations, may be easily applied^18^. Also, two-minute step test (2MST) has already been validated for individuals with obesity^19^. Both are considered field and attractive tests because they represent daily life activities.

Thus, submaximal tests may be appropriate when applied after bariatric surgery, instead of a maximal test, which is a maximum test, since the oxygen uptake (V̇O_2_) of 2MST has been previously correlated with that of incremental treadmill test (ITMT) in individuals with obesity^19^, and the 6MWT promoted V̇O_2_ and heart rate (HR) responses in agreement with ITMT in obese women^20^.

However, to our knowledge, there are no studies that demonstrate the different responses in these tests, as well as which submaximal test would be ideal to apply to individuals with obesity and associated comorbidities or right after a surgical procedure. It is known that 2MST has a shorter test time when compared to the 6MWT, however, 2MST requires a vertical body displacement, differently from the 6MWT. Furthermore, it is still unclear whether body composition and metabolic and lipid profile can compromise functional responses, as observed by the metabolic, ventilatory, cardiovascular, and performance variables in the maximum and submaximal test in individuals’ candidates for bariatric surgery.

In this sense, the primary objective of this study was to analyze whether the body composition, the metabolic and lipid profile influence the maximal and submaximal functional capacity of these individuals. A secondary objective was to verify the metabolic, ventilatory, cardiovascular levels, performance, and perceived effort responses in individuals with obesity in different functional capacity tests (ITMT, 2MST, and 6MWT). We hypothesize that the variables of body composition, metabolic, and lipid profile may be associated with the responses in the tests of functional capacity of these individuals. Furthermore, we believe that 2MST is a test closer to ITMT than 6MWT regarding metabolic, cardiovascular responses, and perceived exertion, being a good alternative when ITMT is not viable, such as after bariatric surgery.

## Materials and methods

### Design and study population

This was a cross-sectional study and followed the Strengthening the Reporting of Observational Studies in Epidemiology (STROBE)^21^. This study was approved by the Federal University of Sao Carlos (UFSCar) Ethics Committee (966.613). The subjects received guidance on the procedures, and all participants gave written informed consent before the study’s initiation. All methods were carried out in accordance with relevant guidelines and regulations (Declaration of Helsinki). The evaluations were conducted in the Cardiopulmonary Physiotherapy Laboratory at the UFSCar, Sao Carlos, SP, Brazil over a 2-year period (2016–2018).

This study recruited individuals with obesity and comorbidities (body mass index (BMI) ≥ 35 kg/m^2^) and individuals with morbid obesity (BMI 40 ≥ kg/m^2^), from both genders and aged between 18 and 50 years, awaiting bariatric surgery in three different medical teams, with a non-random selection of convenience. We contacted them by telephone, explaining our procedures and asking about their interest in participating in our experimental. All selected individuals would perform the Roux-en-Y technique and all patients underwent the same routine protocol before surgery (exams, nutritional and psychological follow-up). The non-inclusion criteria were: patients with orthopedic or neurological impairments that impeded exercise testing; Baecke physical activity questionnaire score greater than 8; history consistent with heart disease; uncontrolled hypertension; uncontrolled and/or insulin-dependent diabetes mellitus; use of beta-blocker; respiratory diseases; the presence of any contraindications to exercise testing^15^; cognitive deficit; difficulty in understanding and/or lack of adherence to the study procedures; users of illicit drugs; pregnant or postmenopausal women.

### Evaluations

All evaluations were performed at the same time of day, to avoid different physiological responses, in a climate-controlled room with relative air humidity between 40–60% and temperature between 22–24 °C. Before the evaluations, the patients performed an adaptation and familiarization with the procedures and received guidance on the tests. The patients were instructed not to ingest any stimulant, and they did not perform strenuous activities within 24 h before the evaluations.

Patients underwent four days of evaluations, and on the first day, an anamnesis with a clinical history was carried out, and a questionnaire on the level of physical activity. On the second day, the individuals were submitted to blood collection, followed by an assessment of body composition. The participants underwent the ITMT on the third day. Respecting a minimum interval of 48 h, patients returned for the fourth visit, and performed 2MST and 6MWT, with a minimum interval of thirty minutes between tests. The tests on the fourth day were randomly chosen to avoid interference in the individuals' performance.

### 1st visit: anamnesis and Baecke's physical activity questionnaire

On the first day, an anamnesis was carried out with the individual's clinical history, with information on past history, medications in use and related comorbidities. The level of physical activity was assessed by information related to occupation, sports activities and leisure habits through the Baecke questionnaire^22^, which was validated and translated for Brazilian adults^23^. This questionnaire consists of a scale from one to five (five representing the most active), with eight questions related to occupation, four addressing sports activities, and four addressing habitual leisure habits. The results are presented with the sum of the points (minimum score of five and maximum of fifteen).

### 2nd visit: blood collection and body composition

Blood samples were collected from the antecubital vein of the upper limb by a qualified professional in a specialized laboratory in the morning and individuals were instructed to fast for 12 to 14 h. Glucose, insulin resistance index by the Homeostasis Model assessment method (HOMA-IR), insulin sensitivity (QUICKI), in addition to the lipid profile: total cholesterol, triglycerides, high density lipoprotein (HDL-c) and low lipoprotein density (LDL-c) were collected.

Subsequently, body composition assessment was performed with the reference standard, already validated for individuals with obesity^24^, the Dual Energy X-Ray Absortiometry (DXA) device (Discovery A, Hologi), and the variables used were bone mineral content (BMC), lean mass (LM) and fat mass (FM), in kilogram. This technique allows estimating body composition as a whole and by body segment.

Patients underwent body composition assessment in the morning, wearing light clothing, barefoot, and without any metal in contact with the body. Everyone was instructed not to perform intense physical exercises on the day before the exam; to fast for four hours before the assessment, and to eliminate urine before the evaluation^25^, as recommended by the manufacturer. The equipment's software automatically defined the body estimation areas, and the results were printed and tabulated in Excel® (Microsoft Excel, 2016).

### 3rd visit: incremental exercise testing

The maximum aerobic capacity assessed by ITMT was based on the Bruce protocol, which shows progressive increases in speed and inclination every three minutes^26^, on a treadmill (Super Inbramed ATL, Rio Grande do Sul, Brasil). The volunteers were encouraged to perform the test until exhaustion. The criteria for stopping/completing the test followed the recommendations of the American Thoracic Society^15^.

The test was performed by two physiotherapists and a physician, enabling potential cardiac arrhythmias detection and also for assistance in case of complications. Volunteers were continuously monitored by a 12-lead electrocardiogram (WinCardio, Microme, Brasília, Brazil) and a cardiofrequencimeter (Polar® S810i, Kempele, Oulu, Finland) fixed on the chest. For the measurement of subjective responses of dyspnea and fatigue in the lower limbs, the Borg scale from 0 to 10 was used^27^, integrating different information in physiological measures of physical performance and work ability. Systolic (SBP) and diastolic (DBP) blood pressure were measured using the auscultatory method. The patients performed two minutes of rest in a sitting position, followed by two minutes in a standing position; after the test, they performed three minutes of active recovery with a speed of 3 km/h and without inclination, followed by three minutes of passive recovery in a sitting position.

The test included gas analysis, and the recording of metabolic and ventilatory parameters was performed using the portable ergospirometry system Oxycon Mobile® (Mijnhardt/Jäger, Würzburg, German) with breath-to-breath measures. All volunteers used a face mask as an interface to collect expired gases during exercise tests.

### 4th visit: six-minute walk test (6MWT) and two-minute step test (2MST)

Both tests were performed according to the recommendations of the American Thoracic Society^28^. Patients were permitted to slow down if necessary and even stop the test for rest. The patients performed two minutes in the sitting position, followed by two minutes in the orthostatic position, and performed six minutes of recovery at the end of each test. Standardized verbal encouragement command was given in both tests. For the 6MWT, patients were instructed to walk as far as possible, without running on a flat surface of 30 m, for six minutes^28^. The 2MST was performed according to the protocol previously described^19^, using a single, portable step, 15 cm high, and without hand rest. The volunteers were instructed to go up and down the step as many times as possible for two minutes (free cadence), with manual recording by the evaluator. An evaluator was responsible for counting up-and-down step cycles (UDS).

As with ITMT, dyspnea perception, and fatigue in the legs as well as HR, SBP, DBP and recording of metabolic and ventilatory parameters were continuously monitored in both tests. In addition, subjective symptoms as dyspnea and fatigue in the lower limbs were also obtained by Borg CR-10.

### Data analysis

For the three exercise tests performed, the peak values were defined as the highest 15-s averaged values. The oxygen uptake (V̇O_2_), carbon dioxide production (V̇CO_2_), minute ventilation (V̇_E_), breathing frequency (BF), and respiratory exchange ratio (RER) were obtained and exported to Excel® (Microsoft Excel, 2016).

### Statistical analysis

A posteriori power analysis was performed using the GPower statistical package (Version 3.1.3 - Franz Faul Universität Kiel, Germany). Considering our study total sample size of 44 individuals, an α error probability of 0.05, and an effect size of 0.50, the statistical power was calculated to be 98%. The data were analyzed using the statistical program SPSS Statistics, Version 20.0, USA. Data were expressed as mean and 95% of confidence interval. Data normality was tested by the Shapiro–Wilk.

The categorical variables were compared with the chi-square test. Between gender differences were evaluated by means of independent-samples t test or Mann–Whitney U test. One-way ANOVA with Tukey post hoc or Kruskall-Wallis were applied to evaluate the difference between tests (ITMT, 2MST and 6MWT). The Pearson's or Spearman’s correlation coefficients were analyzed to investigate the associations between the main variables. The magnitude of the correlation was determined considering the classification of the values of r: 0.00 to 0.19 = none to slight, 0.20 to 0.39 = low, 0.40 to 0.69 = moderate, 0.70 to 0.89 = strong or high, and 0.90 to 1.00 = very high. Besides that, stepwise multiple linear regression analysis was performed to evaluate the best predictors of V̇O_2_ (mL·kg^−1^·min^−1^) and distance walked on ITMT. The variables included in the model were: gender, body composition, metabolic and lipid profile, as well as performance data from the submaximal tests (UDS cycles and distance walked). The statistical significance level was set at p < 0.05.

We had 1 missing value for BMC, LM and FM, and 1 missing for HR on 2MST. These variables were regarded as missing data, not being replaced by an average value.

## Results

**Figure 1:**
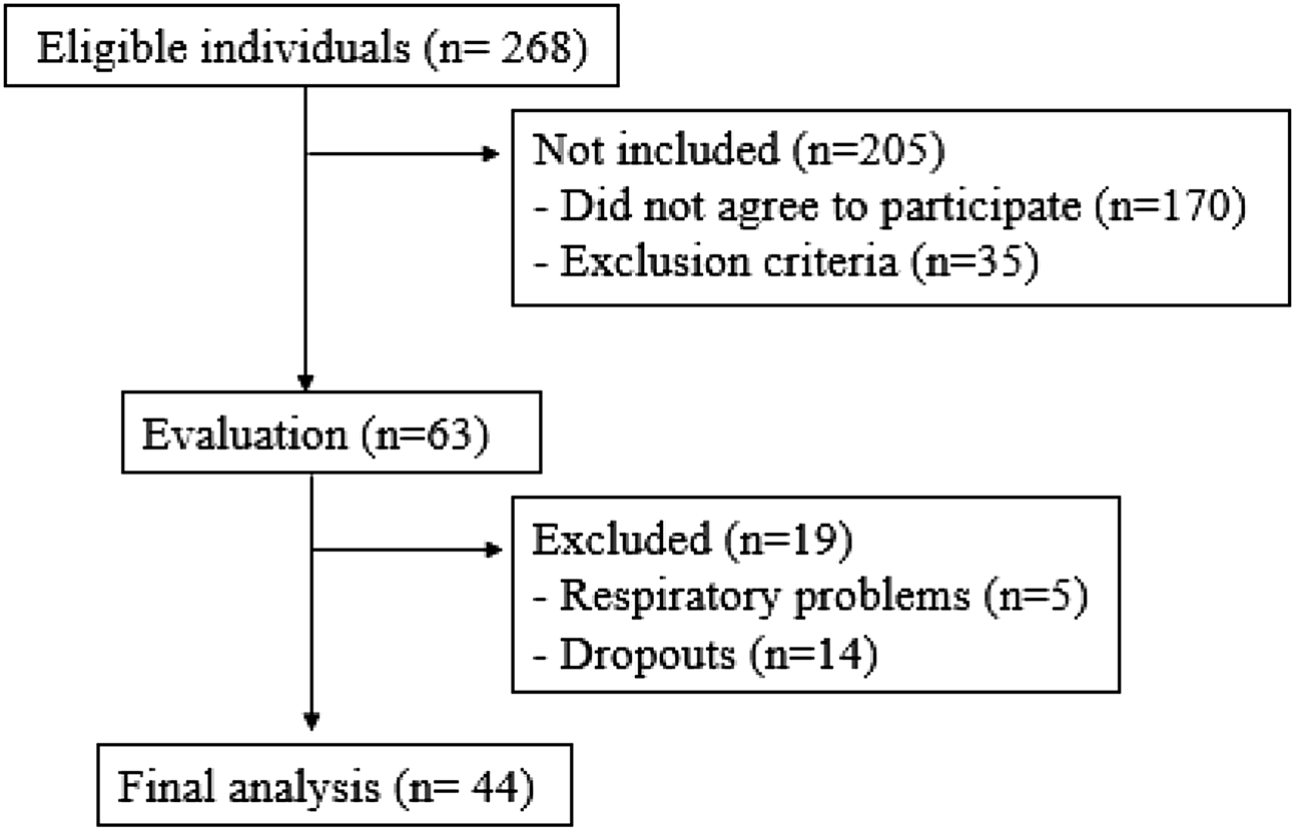
Flow diagram representing sample recruitment.

Figure 1 illustrates the recruitment flowchart for individuals who participated in the study. We initially recruited 268 individuals with obesity, and the final sample was composed by 44 patients.

Table 1 shows patients general characteristics and body composition. Most of the sample was composed of women, as expected, and the most reported comorbidity was arterial hypertension. Considering an absolute total of 15 points, the results of the Baecke questionnaire revealed low physical activity patterns for the evaluated sample. As expected, men had higher values for weight, LM and BMC than women. In addition, men had higher insulin and HOMA values, and lower QUICKI values when compared to women (p < 0.05).

**Table 1:** Risk factors, body composition, metabolic and lipid profile.

Variables	Women (n = 36)	Men (n = 8)	Total (n = 44)
Age, years	36.4 (33.4–39.3)	38.6 (30.7–46.5)	36.3 (33.3–39.4)
Baecke questionnaire	6.7 (6.4–7.2)	6.8 (5.8–7.8)	6.7 (6.4–7.1)
**Comorbidities**			
Type 2 DM, n (%)	5 (13.8)	3 (37.5)	8 (18.2)
Hypertension, n (%)	9 (25)	5 (62.5)	14 (31.8)
Gastroesophageal reflux, n (%)	5 (13.8)	–	5 (11.4)
Thyroid conditions, n (%)	7 (19.4)	–	7 (15.9)
Depressive disorder, n (%)	5 (13.8)	1 (12.5)	6 (13.6)
**Body composition**			
Weight, kg	109.2 (104.2–114.2)	129.5 (116.8–142.1)*	113.4 (107.8–119.1)
BMI, kg/m^2^	40.9 (39.2–42.6)	42.3 (39.0–45.6)	41.0 (39.3–42.7)
LM, kg	51.6 (46.9–53.6)	75.7 (69.4–82.0)*	55.7 (52.2–59.1)
FM, kg	55.0 (51.7–58.3)	51.0 (41.5–60.5)	55.1 (51.6–58.6)
BMC, kg	2.3 (2.2–2.3)	2.8 (2.5–3.0)*	2.4 (2.3–2.5)
**Blood analysis**			
Triglycerides, mg/dL	141.6 (117.0–166.2)	194.2 (142.5–246.0)	150.9 (129.2–172.6)
Total cholesterol, mg/dL	195.9 (184.2–207.6)	219.7 (172.2–267.3)	199.5 (187.5–211.5)
HDL-c,mg/dL	45.1 (42.4–47.8)	41.0 (35.8–46.2)	44.2 (41.9–46.5)
LDL-c, mg/dL	122.5 (113.0–131.9)	140.0 (96.6–183.4)	125.1 (115.0–135.2)
Glucose, mg/dL	96.0 (92.9–99.1)	100.4 (94.3–106.5)	96.6 (93.9–99.3)
Insulin, (mU/L)	14.5 (11.9–17.1)	22.2 (15.9–28.4)*	16.2 (13.3–19.1)
QUICKI	0.32 (0.31–0.33)	0.30 (0.29–0.31)*	0.32 (0.31–0.32)
HOMA-IR	3.5 (2.8–4.2)	5.4 (3.9–7.0)*	3.8 (3.2–4.4)

Data are reported as mean, 95% CI (confidence interval), and percentage. BMI: body mass index, LM: lean mass, FM: fat mass, BMC: bone mineral content, HDL: high-density lipoprotein cholesterol, LDL: low-density lipoprotein, QUICKI: insulin sensitivity index, HOMA-IR: insulin resistance index. *Significant differences between genders.

Table 2 shows that ITMT required greater metabolic, ventilatory and cardiovascular demand, when compared with 6MWT and 2MST. However, only DBP was not significantly different between ITMT and 2MST. In addition, it is worth mentioning that 2MST required a higher V̇O_2,_ and HR, when compared to the 6MWT, with no significant difference in the other variables.

**Table 2:** Responses in incremental treadmill test (ITMT), two-minute step test (2MST) and six-minute walk test (6MWT) at the peak of the tests.

Variables	ITMT (n = 44)	6MWT (n = 44)	2MST (n = 44)
V̇O_2,_ mL·min^−1^	1818.7 (1691.0–1946.5)	1210.2 (1120.3–1300.1)^a^	1379.4 (1289.9–1468.9)^b,c^
V̇O_2,_ mL·kg^−1^·min^−1^	16.1 (15.2–17.0)	10.7 (10.0–11.4) a	12.3 (11.6–13.0)^b,c^
V̇O_2,_ %pred	80.6 (77.5–83.7)	54.3 (51.0–57.7) a	62.6 (58.4–66.7)^b,c^
V̇CO_2,_ mL·min^−1^	2345.6 (2185.8–2505.5)	1169.3 (1076.9–1261.7)^a^	1390.6 (1278.0–1503.3)^b^
RER	1.31 (1.26–1.35)	0.96 (0.93–0.98) a	1.0 (0.96–1.04)^b^
V̇_E,_ L·min^−1^	85.3 (80.9–89.7)	40.4 (37.2–43.6)^a^	46.5 (42.9–50.0)^b^
BF, br·min^−1^	44.9 (42.0–47.9)	31.5 (29.0–34.1)^a^	30.5 (28.3–32.7)^b^
HR, bpm	169.7 (164.5–175.0)	126.0 (122.4–129.5)^a^	139.2 (133.2–145.2)^b,c^
HR, %pred	94.3 (92.0–96.6)	68.5 (66.3–70.6)^a^	75.2 (71.9–78.5)^b,c^
SBP, mmHg	195.1 (187.7–202.5)	163.9 (154.8–173.1)^a^	175.9 (164.7–187.2)^b^
DPB, mmHg	101.8 (98.4–105.3)	96.0 (92.4–99.6)^a^	100.9 (96.1–105.6)
Dyspnea (0–10 score)	7 (1; 10)	2 (0; 10)^a^	3 (0; 7)^b^
Leg fatigue (0–10 score)	3.5 (0; 10)	0.8 (0; 7)^a^	1.5 (0; 7)^b^

Data are reported as mean or median, according to data distribution, and CI (confidence interval). V̇O_2_: oxygen uptake, V̇CO_2_: carbon dioxide production, RER: respiratory exchange rate, V_E_: minute ventilation, BF: breathing frequency, HR: heart rate, *HRmax*: maximal heart rate, SBP: systolic blood pressure, DBP: diastolic blood pressure. Intragroup differences (one-way ANOVA).

^a^ITMT versus 6MWT.

^b^ITMT versus 2MST.

^c^6MWT versus 2MST; p < 0.05.

### Associations between functional tests

A correlation was found between 2MST and the ITMT: UDS cycles x distance walked (r = 0.36; p = 0.01), and between V̇O_2,_ mL·min^−1^ of both tests (r = 0.65; p =  < 0.001). When we compared 6MWT and ITMT we also found a correlation between distance walked (r = 0.33; p = 0.03) and V̇O_2,_ mL·min^−1^ (r = 0.59; p =  < 0.001).

**Figure 2:**
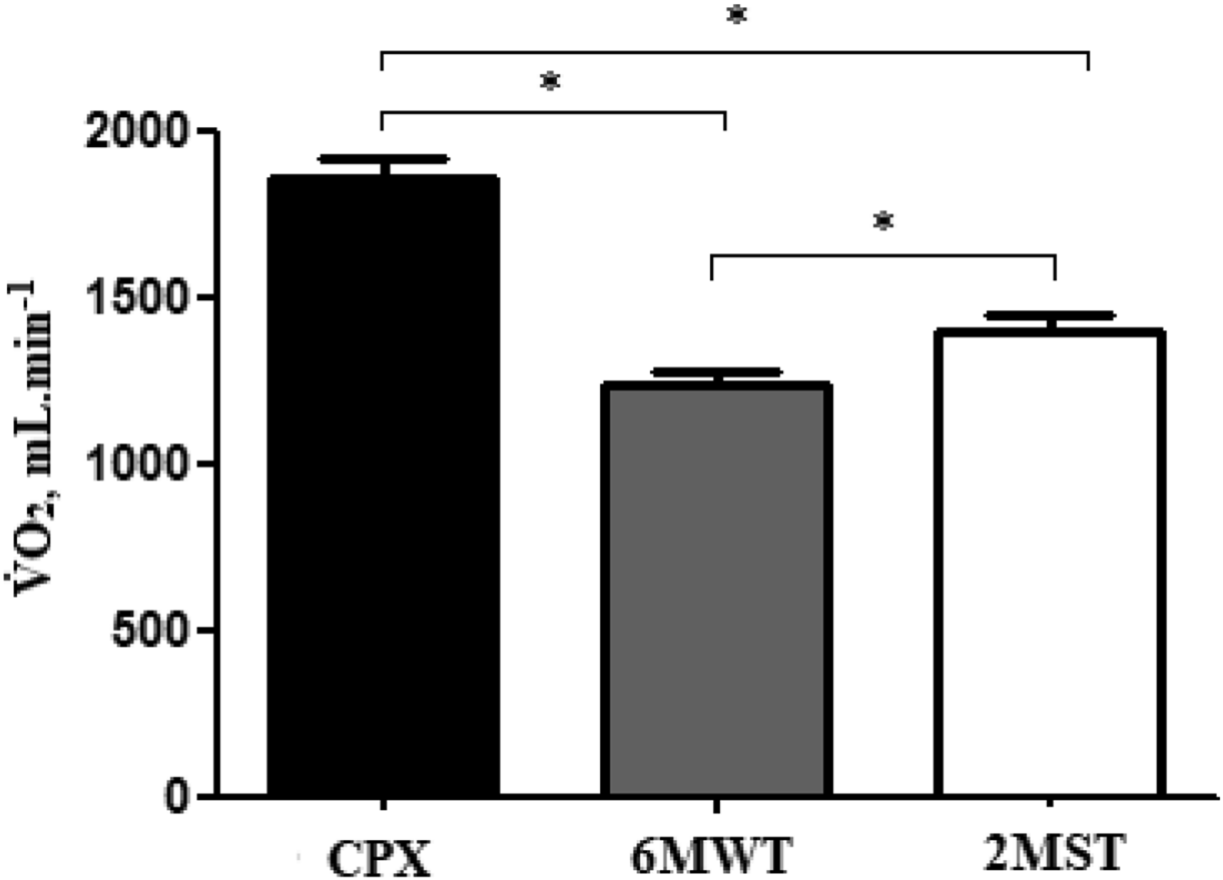
Different responses of V̇O_2,_ mL·min^−1^ on ITMT, 6MWT and 2MST. *p < 0.05.

Moreover, correlations between 6MWT and 2MST were found: UDS cycles × distance walked (r = 0.56; p =  < 0.001) and V̇O_2,_ mL·min^−1^ (r = 0.60; p =  < 0.001). The responses of V̇O_2,_ mL.min^-1^ on ITMT, 6MWT and 2MST are shown in Fig. 2.

### Associations between functional tests and body composition

We found moderate correlation between FM and performance in tests: FM × distance walked on ITMT (r = − 0.48; p = 0.001), FM × Distance walked on 6MWT (r = − 0.32; p = 0.03), without correlation of FM with UDS cycles (r = − 0.22; p = 0.16).

Besides, correlations were found between LM × V̇O_2,_ mL·min^−1^ on ITMT (r = 0.41; p = 0.007), LM × V̇O_2,_ mL·min^−1^ on 6MWT (r = 0.51; p =  < 0.001) and LM × V̇O_2,_ mL·min^−1^ on 2MST (r = 0.35; p = 0.022). Regarding V̇O_2,_ mL·kg^−1^·min^−1^, we can see correlations with FM: FM × V̇O_2,_ mL·kg^−1^·min^−1^ on ITMT (r = − 0.43; p = 0.003), FM × V̇O_2,_ mL·kg^−1^·min^−1^ on 6MWT (r = − 0.42; p = 0.005) and FM × V̇O_2,_ mL·kg^−1^·min^−1^ on 2MST (r = − 0.37; p = 0.01).

**Figure 3:**
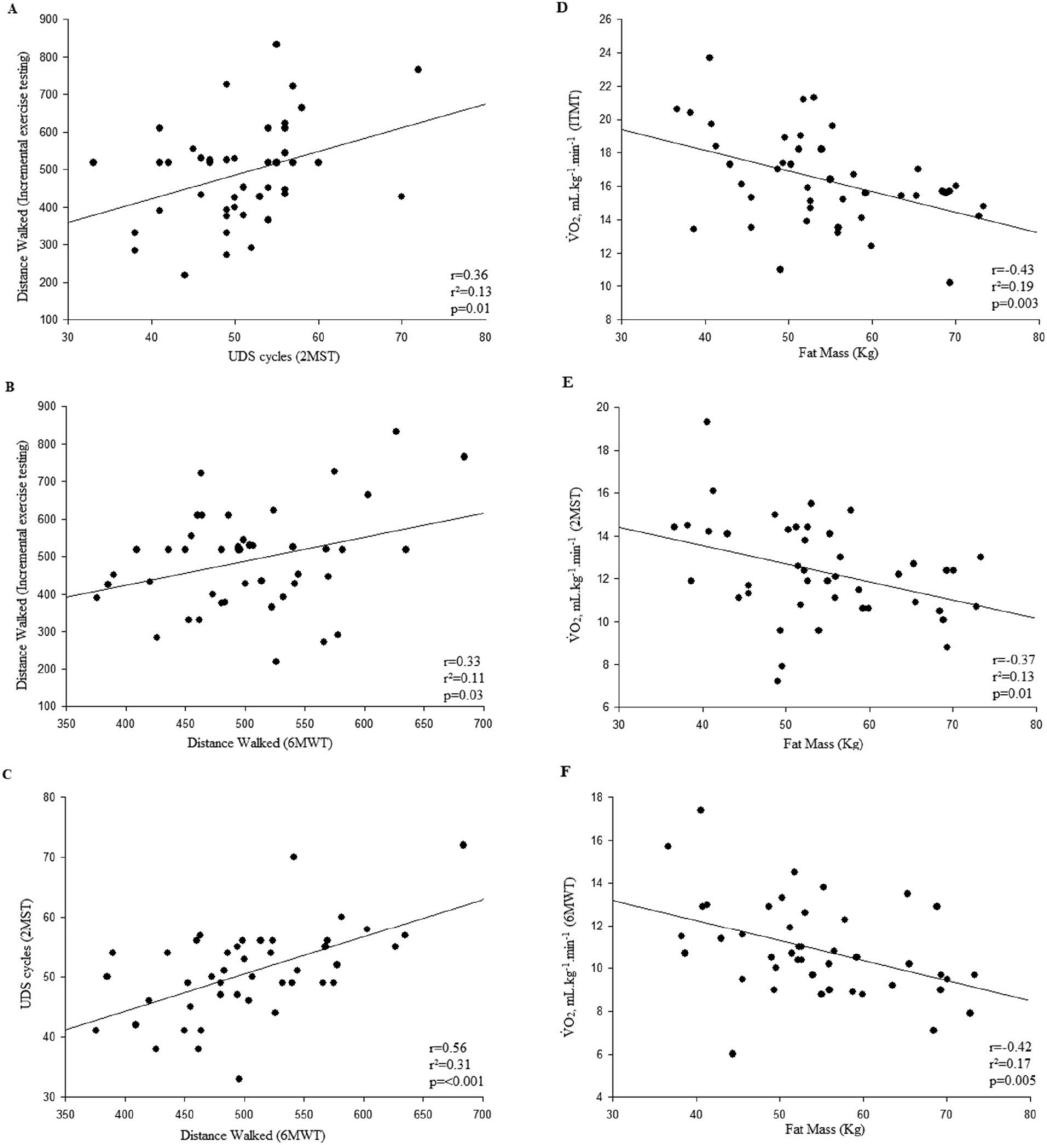
(**A**) Correlation between distance walked on ITMT and UDS cycles on 2MST; (**B**) Correlation between distance walked on ITMT and on 6MWT; (**C**) Correlation between UDS cycles on 2MST and distance walked on 6MWT; (**D**) Correlation between V̇O_2,_ mL·kg^−1^·min^−1^ on ITMT and fat mass; (**B**) Correlation between V̇O_2,_ mL·kg^−1^·min^−1^ on 2MST and fat mass; (**C**) Correlation between V̇O_2,_ mL·min^−1^ on 6MWT and fat mass.

Correlations of performance between tests and body composition are shown in Fig. 3.

### Estimation models

In the stepwise multiple linear regression analysis for V̇O_2_ at the peak of ITMT, FM and LDL were considered significant in the model (p < 0.05), which explains 30% of the V̇O_2_ variance in the test. The model is described in Table 3. Regarding the distance walked, the model considered the FM, glucose and UDS cycles performed in the 2MST, which were able to explain 42% of the variance of the distance walked in the ITMT, as shown in Table 4. The gender was not significant variable to include in the predictive models.

**Table 3:** V̇O_2_ peak (mL·kg^−1^·min^−1^) predictive model considering total fat mass and LDL.

Variables	Coefficient	Std. Error	*p* value
**R**^**2**^** = 0.299**			
Constant	19.782	2.450	< 0.001
FM	− 0.127	0.037	0.002
LDL	0.028	0.011	0.017

FM: fat mass, LDL: lipoprotein density.

**Table 4:** Distance walked predictive model based on total fat mass, glucose and UDS cycles during 2MST.

Variables	Coefficient	Std. Error	*p* value
**R**^**2**^** = 0.419**			
Constant	1016.789	225.380	< 0.001
FM	− 4.992	1.707	0.006
Glucose	− 5.416	1.875	0.006
UDS cycles	5.281	2.215	0.022

FM: fat mass.

## Discussion

### Main findings

This study presents some important results for this population: (1) ITMT elicited greater metabolic, ventilatory and cardiovascular demand when compared to the 6MWT and 2MST in patients with obesity; (2) even with the time difference between the 6MWT and the 2MST, and the activity performed between them (horizontal and vertical, respectively), the 2MST, imposed a greater chronotropic (HR) and metabolic (V̇O_2_) demand, since these variables differed from each other, and that did not seem noticeable from a ventilatory, blood pressure, dyspnea and leg fatigue point of view; (3) body composition (LM and FM) is able to influence the functional capacity of individuals with obesity, observed by the responses in the three different tests presented, and (4) the estimation model based on FM and LDL can explain 30% of V̇O_2_ variance at the peak of the ITMT, and the model based on the FM, glucose and UDS cycles, is able to explain 42% of the distance walked in the ITMT.

### Comparison between cardiovascular and metabolic ventilatory variables between maximal and submaximal tests

The maximal incremental cardiopulmonary exercise may have limitations in its performance because it needs an adequate space and a highly trained team (with a doctor), and it would not be feasible to be applied mediately after a surgical procedure, due to the high demand required. Moreover, individuals with obesity and comorbidities have a substantial limitation in activities due to their inability to perform high-intensity exercise^29^. The use of the exercise test with the assessment of cardiopulmonary variables is extremely important, since general health status better correlates with exercise tolerance than with measures at rest^15^. In addition, this evaluation provides us with valuable information, which optimizes the appropriate intensity finding for an intervention program, and, in this way, the systems' integrative responses can be evaluated in submaximal tests, assisting in clinical decision making^30^.

Thus, activities where the level of effort is related to the effort required for day-to-day activities seem adequate to assess functional capacity. Da Costa and collaborators^31^ compared the 6MWT and the six-minute step test (6MST) and observed that both are safe and produce submaximal efforts in healthy and sedentary individuals. Nonetheless, they concluded that 6MST requires greater oxygen demands due to different body movements when compared to 6MWT. Still, 6MST is not always a submaximal test when used in some populations, as it is known that the stress to perform activities that require vertical body displacement causes greater fatigue^32^. In this sense, some authors suggest that a shorter step test may be appropriate in individuals who have cardiopulmonary or musculoskeletal limitations, since the frequency of UDS cycles may be constant after the second or third minute^31–33^.

Pessoa and collaborators^34^ demonstrate that the 2MST is a sensitive and reliable test when analyzing the functional capacity in individuals with severe chronic obstructive pulmonary disease, as it is a short-term test and was sufficient to cause metabolic, ventilatory, cardiovascular and of effort perception. In addition, the 2MST also allows a reliable and safe assessment in individuals with heart failure, as it demands a greater effort, which the 6MWT did not demonstrate. Besides that, the authors performed the same tests performed in the present study (ITMT, 2MST and 6MWT), and it is highlighted that V̇O_2_ peak of 2MST correlated with ITMT, corroborating our result, suggesting that 2MST can be used for evaluation of the global integrated response to exercise, in a way correlated to the ITMT, for example^35^.

Therefore, 2MST proves to be a viable option for several populations, and when compared to 6MWT in the current study, it shows that even with a shorter testing time and a different activity, it required a greater chronotropic (HR) and metabolic (V̇O_2_) demand, with no difference in dyspnea and leg fatigue perception. Unlike other studies that carry out walking in place during 2MST (the individual needs to raise the knees, one at a time, to the height between the middle of the patella and the iliac crest as many times as possible)^35,36^, we performed the test on an ergometer in order to obtain the vertical and horizontal displacement of the patient, quite similarly to the 6MST.

In comparison to ITMT, 2MST differed from all variables, except for DBP. However, it presented a moderate correlation in the test performance, and in V̇O_2_. Although 6MWT is widely used and practical on a day-to-day basis, we could observe in this study that the demand is much lower, even with a test time longer than 2MST, in addition to needing a space (30 m corridor) for its realization.

### Relationship between fat mass and functional capacity obtained in the maximal and submaximal tests

It is clear in the literature that obesity causes much damage to health^37,38^, and that these individuals have significant limitations when performing physical exercise, as this has been clearly investigated^6,39^ and compared with eutrophic individuals^40,41^. Excess body mass affects motor function, causing individuals with obesity to have greater energy expenditure^42,43^. Physiologically, it is more difficult for the individual with obesity to do the same amount of work as a eutrophic person, since the excess fat does not contribute to the work performed, hindering performance^42^. Uranga et al.^11^ observed that many of the risk factors that are related to obesity depend mainly on the distribution of fat, since the adipose tissue intensifies the state of inflammation^44,45^. In our study, it was possible to observe that FM negatively affected V̇O_2_peak in both maximum and submaximal tests.

Excessive infiltration of fat in the muscle tissue of the lower limbs may explain the impairment in performance and difficulty in daily physical functions^11,43,46^, and it has already been associated with metabolic disorders, such as insulin resistance, and changes in glucose metabolism^43^. In this sense, impairment of motor function in individuals with obesity seems to depend on mechanical and metabolic factors that significantly reduce global motor performance^11^.

### Predictors of functional capacity obtained in the maximal and submaximal tests

In the multiple regression analysis of V̇O_2_ in ITMT, FM and LDL were considered. In addition to the implications of body fat for performance already mentioned in the previous paragraphs, individuals with obesity have high levels of LDL^47^ and elevations of this lipoprotein have already been associated with a high risk of developing cardiovascular disease^48^. The guidelines of the Adult Treatment Panel III^49^ recommend that the ideal LDL level be < 100 mg/dL, and in our study, we observed that individuals have an average of 125.1 mg/dL. Although not evaluated in the present study, endothelial dysfunction, a consequence of the formation of metabolic products derived from lipids, hormones and pro-inflammatory cytokines, is present in individuals with obesity^50,51^. There are several related mechanisms attributed to the progression of endothelial dysfunction in individuals with obesity, and it includes increased levels of LDL and triglycerides, increased oxidative stress, elevated levels of inflammatory factors and unbalanced hemodynamic activities^51^, and it can be a factor that may explain the contribution of LDL in the predictive model.

In the analysis for the distance walked in the ITMT, however, it was also considered the FM, the performance in the 2MST (obtained through the UDS cycles), in addition to the glucose. The 2MST UDS cycles correlated with the distance walked in the ITMT, and the responses in the 2MST seem to be closer to the ITMT, instead of the 6MWT. Obesity is also associated with dyslipidemia, which can be explained by the expansion of visceral adipose tissue^44^. Insulin resistance, diabetes mellitus, and impaired glucose metabolism are commonly found in individuals with obesity^10,52^, and are associated with poor quality of skeletal muscles^43^. Additionally, it is worth mentioning that the individuals in our study showed altered values in the HOMA and QUICK tests (valid estimates for insulin resistance)^53^.

In view of all the aforementioned changes, there are several benefits of physical exercise for individuals with obesity^38,54^, for atherogenic reduction^55,56^, in the improvement of endothelial dysfunction^50,57^, with potent cardiovascular effects^51^ and in reducing mortality^56^. However, the prescription of physical exercises often becomes a challenge for the individuals with obesity^58^. In this sense, effective methods for assessing functional capacity must be applied, such as for prognostic purposes before bariatric surgery^16^, and even after the surgical procedure, to start an intervention program.

In this sense, individuals with obesity, often with comorbidities, are candidates for bariatric surgery, and a test for the assessment of effective, simple and quick functional capacity can be beneficial in a preoperative moment, and right after the surgical procedure, since it can help to monitor possible functional declines and assist in rehabilitation strategies, as it has been carried out in the studies of our group^59,60^.

### Limitations of study

The present study has limitations that must be considered. According to our exclusion criteria, these findings cannot be extrapolated to individuals who have neurological, cardiac, respiratory disorders, etc. Unfortunately, we did not evaluate the endothelial function in this study, in addition to the infiltration of intramuscular fat, which should be performed through magnetic resonance imaging. Our sample consisted mostly of women, however, gender was not significant in the predictive models in the statistical analysis, so we can consider that this was not a confounding factor. Nonetheless, the population of obese patients undergoing bariatric surgery in Brazil is mostly women^61^, which was reflected in our sample. In addition, we consider it appropriate that future studies investigate these findings in a more balanced way.

## Conclusions

We conclude that obesity and changes in body composition and in the metabolic and lipid profile are capable of compromising functional capacity. As expected, ITMT generated greater metabolic, ventilatory and cardiovascular demand, when compared to 2MST and 6MWT. In addition, both submaximal tests showed associations with ITMT in performance and V̇O_2_. However, 2MST imposed greater metabolic (V̇O_2_) and chronotropic (HR) demand when compared to the 6MWT. Our findings may indicate the importance of performing submaximal tests that consider horizontal and vertical displacements as important tools in the clinical evaluation of patients who will undergo bariatric surgery, in order to assess the activities of daily living, as well as the effects of interventions with physical exercises after surgical procedure.

## Data Availability

The datasets used and/or analyzed during the current study are available from the corresponding author on reasonable request.
